# Troponin T but not C reactive protein is associated with future surgery for aortic stenosis: a population-based nested case-referent study

**DOI:** 10.1136/openhrt-2020-001325

**Published:** 2020-10-13

**Authors:** Anders Holmgren, Johan Ljungberg, Johan Hultdin, Bengt Johansson, Ingvar A Bergdahl, Ulf Näslund, Stefan Söderberg

**Affiliations:** 1 Public Health and Clinical Medicine, Cardiac Surgery, Umeå University, Umeå, Sweden; 2 Public Health and Clinical Medicine, Medicine, Umeå University, Umeå, Sweden; 3 Medical Biosciences, Clinical Chemistry, Umeå University, Umeå, Sweden; 4 Biobank Research, Umeå University, Umeå, Sweden

**Keywords:** aortic valve disease, epidemiology, risk factors, surgery-valve

## Abstract

**Aims:**

High-sensitivity troponin T (hs-TnT) and high-sensitivity C reactive protein (hs-CRP) may convey prognostic information in patients with aortic stenosis (AS). This study evaluated if hs-TnT and hs-CRP associate with myocardial mass, and risk of future surgery for AS.

**Methods:**

In total, 336 patients (48% women) with surgery for AS with previous participation in large population surveys were identified. Preoperatively, myocardial mass and the presence of coronary artery disease (CAD) were assessed. Two matched referents were allocated for each case, and hs-TnT and hs-CRP were determined in stored plasma from the baseline survey. Conditional logistic regression analysis was used to estimate the risk (OR (95% CI)) related to one (natural logarithm) SD increase in hs-TnT and hs-CRP. Kaplan-Mayer and Cox regression analyses were used to evaluate time to surgery.

**Results:**

Median age (IQR) was 59.8 (10.3) years at survey, and median time between survey and surgery was 10.9 (9.3) years. Hs-TnT was independently associated with surgery for AS (1.24 (1.06–1.44)) irrespective of CAD, whereas Hs-CRP was not (1.05 (0.90–1.22)). Elevated hs-TnT levels at survey associated with shorter time to surgery (p<0.001), and with increased myocardial mass (p=0.002). Hs-CRP did not associate with time to surgery or with myocardial mass.

**Conclusions:**

Hs-TnT—but not hs-CRP—was associated with increased risk of—and shorter time to—future surgery for AS. Hs-TnT associated with myocardial mass at surgery which indicates that hs-TnT could be a potential biomarker for determining intervention.

**Key questions**

**What is already known about this subject?**

- To the best of our knowledge, the significance of elevated troponin T (TnT) levels years before surgery for aortic stenosis has not described previously.

**What does this study add?**

- These findings are novel and enrich the knowledge about predictors for aortic stenosis. We also show that aortic stenosis has a least two phenotypes (with and without concomitant coronary artery disease). To better understand the risk factors behind aortic stenosis and the pathophysiology, the phenotype must be considered, which should help us to better design clinical trials for the prevention of aortic stenosis.

**How might this impact on clinical practice?**

- We suggest that high-sensitivity TnT should be evaluated as could be a potential biomarker for determining intervention for aortic stenosis.

## Introduction

Degenerative aortic stenosis (AS) is the most common valve disorder that requires surgery in adults in developed countries and the prevalence is expected to increase as a consequence of increased life expectancy. Once symptoms occur, the prognosis of untreated AS is poor and surgical aortic valve replacement (AVR) or transcatheter aortic valve implantation are the only available therapeutic options. The pathology of the aortic valve lesions involves active atheromatous and inflammatory processes sharing some histological similarities with atherosclerosis. Furthermore, AS and atherosclerosis have similar associations with traditional cardiovascular risk factors and coronary artery disease (CAD) is a common finding in patients requiring surgery for AS. This suggests that AS represents an atherosclerosis-like process involving the aortic valve.^1 2^ AVR is indicated as soon as symptoms occur,^3^ but there is a rather weak correlation between the onset of symptoms and the severity of AS which makes it difficult to decide the optimal time for surgical intervention.

AS is associated with the development of left ventricular hypertrophy. Established left ventricular hypertrophy at time for surgery is a predictor for worse outcome after surgery.^4^ In the early stages of AS, increased left ventricular mass is found in 20% of patients, compared with 70% in patients with severe AS.^5^

There is no established biomarker for the early development of increased left ventricular mass, even though several markers have been proposed.^6^ According to current guidelines, there is a need to explore early markers of left ventricular dysfunction related to prognosis.^3^ An objective biomarker could improve risk stratification in patients with AS and facilitate the identification of patients who could benefit from an earlier intervention. High-sensitivity troponin T (hs-TnT) levels at AVR relates to prognosis,^7 8^ but has not yet been clinically established as a prospective marker years before the AVR. It has also been documented as an independent marker of increased risk of cardiovascular disease (CVD) in the general population.^9^ Elevated C reactive protein (CRP) levels are associated with the atherosclerotic process,^10^ but the results from previous cross-sectional studies regarding AS have been divergent.^11–13^

In this nested case-reference study, we hypothesised that elevated levels of high-sensitivity CRP (hs-CRP) and hs-TnT were associated with increased risk for, and shorter time to, future surgery for AS. Furthermore, we hypothesised that preoperative levels of hs-CRP and hs-TnT were associated with myocardial mass.

## Material and methods

### Study population

**Figure 1:**
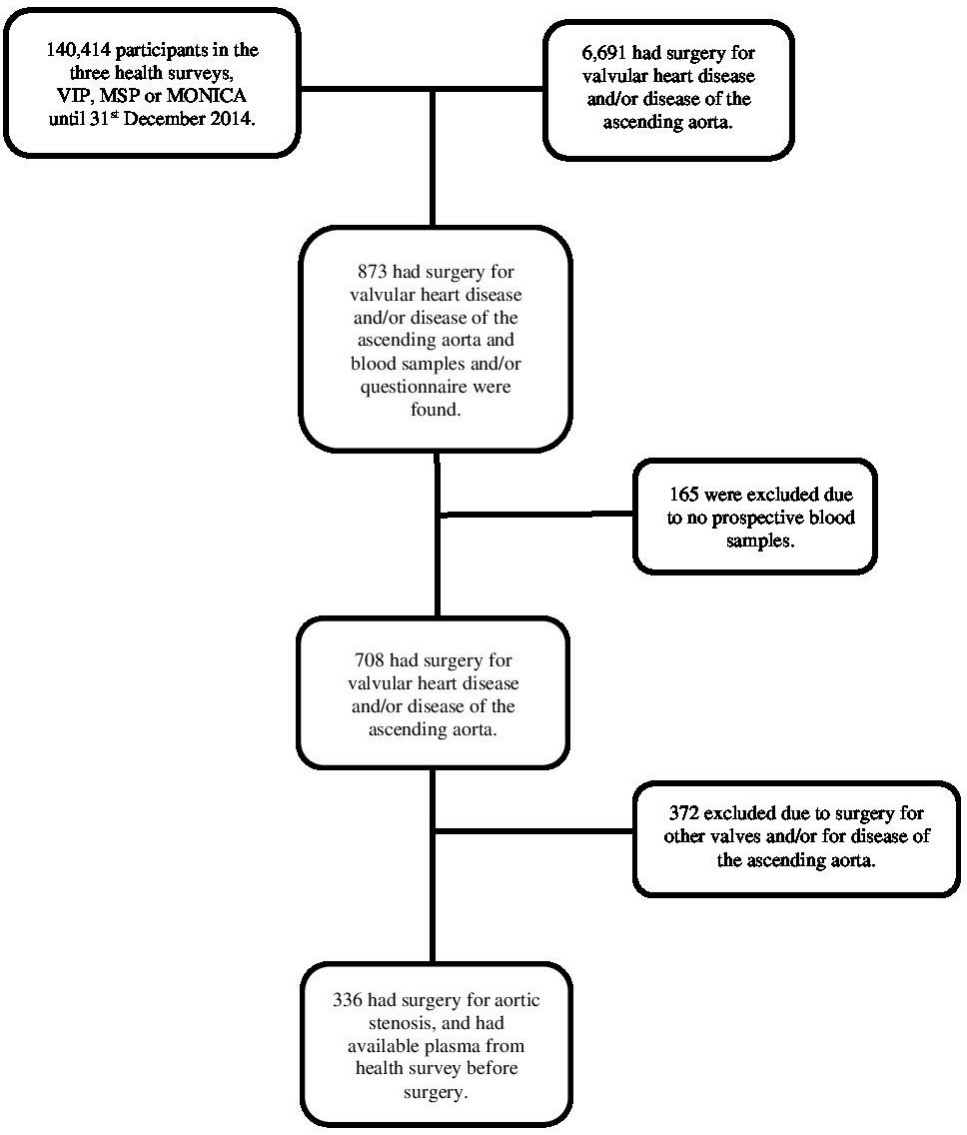
Flow chart showing the selection of cases within the NSHDS cohort. MONICA, MONItoring of trends and determinants in CArdiovascular diseases; MSP, Mammary Screening Programme; NSHDS, Northern Sweden Health and Disease Study; VIP, Västerbotten Intervention Programme.

In this nested case-referent study, cases were patients with AVR and previous participation in large-population-based surveys. During a 27-year period (March 1988 to December 2014), 6691 patients underwent surgery for valvular heart disease and/or disease of the ascending aorta at the Department of Cardiothoracic Surgery, Umeå University Hospital, Umeå, Sweden. Of these, 873 patients had before surgery participated in the Northern Sweden Health and Disease Study which is based on three population-based health studies, the Västerbotten Intervention Programme (VIP), the MONItoring of trends and determinants in CArdiovascular diseases (MONICA) project and the Mammary Screening Programme (MSP). In total, 336 of them had an AVR due to AS with concomitant coronary artery by-pass surgery (CABG) if indicated, and 237 had previously participated in VIP, 37 in MONICA and 62 in MSP. The selection process of cases is summarised in figure 1.

VIP is an ongoing community intervention programme with the aim of preventing CVD and diabetes in the county of Västerbotten.^14^ In this programme, all county residents, at the ages of 30 (until 1995), 40, 50 and 60 years, were asked to participate in a health survey and receive health counselling at their primary healthcare centre. MONICA enrolment involved asking randomly selected individuals in the counties of Västerbotten and Norrbotten (530 000 inhabitants), to participate in a health survey.^15^ The participants were 25–74 years of age. The MSP cohort comprised women that attended routine mammography screenings.^16^ Taken together, these three surveys included 140 414 participants up to December 2014, which reflected participation rates of 65%–75%. All participants were asked to donate blood for future research.

For each case, two referents were matched with their case for sex, age (±2 years), type of survey, date of health survey (±4 months) and geographical area. Referents or cases with a history of myocardial infarction (MI) or cancer prior to survey were not excluded.

### Perioperative characteristics

From hospital files, we identified preoperative and perioperative information such as medical history and surgical information about the nature of valvular disease and type of valvular intervention, and echocardiograms and coronary angiograms were identified. A preoperative echocardiogram was performed in all cases, and left ventricular mass was possible to calculate in 76% of all cases using Deveraux formula.^17^ According to established clinical practice, all except one patient (99.7%) underwent a coronary angiogram, and any atheromatosis was regarded to indicate CAD (found in 60% of all cases with coronary angiograms).

Patients with CAD were more often men and were older at both survey and surgery. They more often had a history of a previous MI and coronary bypass surgery. They had higher systolic blood pressure, and their aortic valves were less stenotic with lower gradients (online supplemental table 1). Referents did not have any echocardiogram or coronary angiogram.

10.1136/openhrt-2020-001325.supp1 Supplementary data

### Baseline clinical examinations and biochemical analysis

Clinical examinations and biochemical analyses performed at baseline have been thoroughly described in our previous paper.^18–20^ Participants in VIP and MONICA were asked to complete a health questionnaire regarding their living conditions and cardiovascular risk factors. Subjects were categorised as smokers (including current daily smokers and ex-smokers) or never-smokers.

An oral glucose tolerance test was performed routinely in the VIP, in 60% of MONICA participants, but not in the MSP. Glucose tolerance categories were defined according to WHO guidelines.^21^ Anthropometry and blood pressure measurements were performed as previously described,^19^ and hypertension was defined as a systolic blood pressure ≥140 mm Hg, diastolic blood pressure ≥90 mm Hg and/or reported use of antihypertensive medication.

Plasma samples were obtained after fasting for a minimum of 4 hours (extended to 8 hours, after 1992). The samples were stored in a blood bank at –80°C until analysis.

In 2017, the following analyses were performed on a Cobas 8000 modular analyzer.

Hs-CRP: The reagents employed were CRPL3 (catalogue No. 05172373190; Roche Diagnostics, Basel Switzerland). Lowest level of detection was 0.3 mg/L. CRP is traceable to CRM 470 (CRPL3 2011–01, V3). The total coefficients of variation were 1.5% and 1.9% at levels of 8 and 47 mg/L, respectively.

Hs-TnT: The reagents employed were TnT hs STAT (catalogue No. 05092728190) Roche Diagnostics, Basel, Switzerland). Lowest level of detection was 3 ng/L. The total coefficients of variation were 5.4% and 2.0% at levels of 29 and 2362 ng/L, respectively.

Apolipoprotein A1(Apo A1) and Apolipoprotein B (Apo B): The reagents employed were Tina-quant Apo A1 and B (catalogue Nos. 03032566122 and 03032574122, respectively, both V.2). Apo A1 and Apo B were standardised to reference standards IFCC SP1-01 and SP3-07, respectively. The total coefficients of variation were Apo A1 3.42% and 2.18% at levels of 0.86 and 1.45 mg/L, respectively; and Apo B 1.93% and 2.19% at levels of 1.0 and 1.8 mg/L, respectively.

### Statistical analysis

Continuous data were assessed for normal distribution with formal tests and by visual evaluation, and data were transformed to the natural logarithm (ln) scale to correct for skewed distributions if needed. The (ln) z-scores were calculated to make different scales comparable, and was done separately for men and women. As a conservative approach, missing values were replaced with the median value obtained among the referents, calculated separately for men and women. The dataset with replaced missing values enabled the usage of the entire dataset. Continuous variables were also categorised into quartiles, based on the distribution of the referent values. Missing values were treated as a separate category and were not included in the tables.

Data are presented as the (geometric) mean with 95% CIs, Student’s t-tests for independent groups were used to analyse differences in means between cases and referents. Within strata, the cases and referents had the same follow-up times in this nested case-referent study. Therefore, we estimated OR and 95% CI with logistic regression analyses (rather than Cox regression) and the conditional maximum likelihood routine designed for matched analysis. The influence of studied variables on future surgery for AS was tested in univariable and multivariable models. Model 1 included hs-CRP and hs-TnT, model 2 included Apo B/A1 ratio, hypertension (yes/no), glucose intolerance (yes/no) and smoking (present or past/never), and in the final model, body mass index (BMI) was added to model 2. The analyses were stratified for sex, the time interval between survey and surgery (less than 5 years or 5 years and more), and the presence of any CAD on the preoperative angiogram. Finally, in separate analyses, we excluded the MSP cohort since several cardiovascular risk factors were not registered in MSP.

In cases, Kaplan-Meier with log rank tests and Cox regression survival analyses were used to evaluate the effect of hs-CRP and hs-TnT on time to surgery. The multivariable model included sex and age at survey.

The associations between the dependent variables hs-CRP and hs-TnT and the independent variables left ventricular mass and the gradient over the aortic valve adjusted for sex, age at survey and time to surgery were tested in a linear regression model which included an interaction term between left ventricular mass and gradient over the aortic valve.

All calculations were performed with the SPSS V.26 (IBM).

## Results

Altogether 336 patients (48% women) received an AVR due to AS. In 84% of patients, the primary indication was AS; the remaining 16% received aortic valvular surgery combined with another primary intervention, such as CABG (10%) or surgery for ascending aortic disease (5%). Median age (IQR (IQR)) at survey was 59.8 (10.3) years and 68.3 (12.7) at surgery. The median time between survey and surgery was 10.9 (9.3) years. At survey, individuals with future surgery for AS had higher BMI, higher blood pressure, higher total cholesterol, higher Apo B and a higher Apo B/A1 ratio (table 1). They also more often had a diagnosis of hypertension and glucose intolerance. Circulating levels of hs-TnT were higher among cases than among referents, whereas circulating levels of hs-CRP did not differ. After stratification for the presence of CAD, hs-TnT levels were higher in cases irrespective of CAD, whereas hs-CRP levels were higher only in cases with concomitant CAD.

**Table 1:** Subject characteristics at baseline survey

	N (referents/cases)	Referents	Cases	P value
Women, %	671/336	48 (44–52)	48 (43–53)	Matched
Age at survey, year	671/336	56.7 (56.0–57.3)	56.7 (55.8–57.6)	Matched
Age at surgery, year	–/336	–	67.2 (66.3–68.2)	
BMI, kg/m^2^	655/322	26.1 (25.8–26.4)	26.9 (26.4–27.4)	0.01
Hypertension, %	545/269	49.2 (45.0–53.4)	61.0 (55.1–66.8)	0.001
Systolic blood pressure, mm Hg	545/270	136 (134–137)	138 (136–141)	0.04
Diastolic blood pressure, mm Hg	545/269	85 (84–85)	86 (85–87)	0.05
Glucose intolerance, %	490/242	19.8 (16.3–23.3)	26.4 (20.8–32.0)	0.05
Smoker, %	531/258	53.7 (49.4–57.9)	59.7 (53.7–65.7)	0.11
Total cholesterol, mmol/L	535/265	6.2 (6.1–6.3)	6.4 (6.2–6.5)	0.05
Apolipopotein B, g/L*	647/310	1.09 (1.07–1.11)	1.13 (1.10–1.16)	0.05
Apolipoprotein A1, g/L*	647/309	1.41 (1.40–1.43)	1.40 (1.37–1.42)	0.25
Apolipoprotein B/A1, ratio*	647/309	0.77 (0.76–0.79)	0.81 (0.79–0.84)	0.01
hs-CRP, mg/L*				
All	646/310	1.2 (1.1–1.3)	1.3 (1.2–1.5)	0.07
Men	334/155	1.0 (0.9–1.0)	1.2 (1.0–1.3)	0.06
Women	312/155	1.2 (1.1–1.4)	1.6 (1.4–1.8)	0.006
Surgery <5 years after survey	146/69	1.2 (1.0–1.3)	1.4 (1.2–1.7)	0.08
Surgery ≥5 years after survey	500/241	1.1 (1.0–1.2)	1.3 (1.2–1.44)	0.006
No CAD	256/128	1.1 (1.0–1.2)	1.2 (1.0–1.4)	0.33
CAD	388/181	1.1 (1.0–1.3)	1.4 (1.3–1.6)	0.005
hs-TnT, ng/L*				
All	647/310	4.4 (4.2–4.5)	4.8 (4.5–5.1)	0.01
Men	335/155	4.6 (4.4–4.8)	5.2 (5.0–5.6)	<0.001
Women	312/155	3.8 (3.7–3.9)	4.0 (3.8–4.3)	0.09
Surgery <5 years after survey	146/69	4.7 (4.4–5.1)	6.4 (5.7–7.3)	<0.001
Surgery ≥5 years after survey	501/241	4.1 (4.0–4.2)	4.3 (4.1–4.5)	0.08
No CAD	256/128	3.9 (3.8–4.1)	4.5 (4.2–4.8)	<0.001
CAD	389/181	4.6 (4.4–4.8)	5.0 (4.7–5.3)	0.04

Glucose intolerance, impaired fasting glucose or impaired glucose intolerance or diabetes mellitus; hypertension, systolic blood pressure ≥140 and/or diastolic blood pressure ≥90 and/or antihypertensive treatment; smoker, present or previous smoker.

Values shown are numbers, means (*geometric) and proportions with 95% CIs; p values were based on the Student’s t-test.

BMI, body mass index; CAD, coronary artery disease; hs-CRP, high-sensitivity C reactive protein; hs-TnT, high-sensitivity troponin T.

In the univariable analysis, elevated levels of hs-TnT, expressed as a 1-SD increase in (ln) hs-TnT was associated with surgery for AS (OR (95% CI)) (1.24 (1.07 to 1.43)) (table 2). Similarly, hs-TnT levels corresponding to the two highest quartiles were associated with future AVR. Levels of hs-CRP expressed as a 1 SD increase in (ln) hs-CRP, were not associated with future surgery for AS (1.13 (0.98 to 0.30)). However, an association was seen in those with CAD but not in those without CAD.

**Table 2:** Univariable analyses

	hs-CRP					hs-TnT				
	Referents	Cases	All, OR (95% CI)	No CAD, OR (95% CI)	CAD, OR (95% CI)	Referents	Cases	All, OR (95% CI)	No CAD, OR (95% CI)	CAD, OR (95% CI)
Q1	164	70	1.00	1.00	1.00	248	92	1.00	1.00	1.00
Q2	163	58	0.86 (0.57 to 1.30)	0.54 (0.28 to 1.04)	1.20 (0.69 to 2.08)	77	39	1.54 (0.96 to 2.47)	1.17 (0.56 to 2.43)	1.88 (1.00 to 3.54)
Q3	160	89	1.35 (0.91 to 2.01)	0.96 (0.52 to 1.74)	1.85 (1.08 to 3.18)	161	81	1.65 (1.10 to 2.47)	1.46 (0.79 to 2.69)	1.88 (1.08 to 3.26)
Q4	160	93	1.40 (0.94 to 2.06)	0.99 (0.55 to 1.80)	1.79 (1.05 to 3.05)	161	98	2.20 (1.43 to 3.40)	2.09 (1.04 to 4.17)	2.29 (1.29 to 4.07)
Z-scores										
All (missing)	646	310	1.13 (0.98 to 1.30)	0.97 (0.78 to 1.21)	1.24 (1.03 to 1.50)	646	310	1.27 (1.10 to 1.47)	1.40 (1.07 to 1.83)	1.20 (1.01 to 1.43)
All	671	336	1.13 (0.98 to 1.30)	0.98 (0.79 to 1.21)	1.24 (1.03 to 1.48)	671	336	1.24 (1.07 to 1.43)	1.39 (1.06 to 1.82)	1.17 (0.98 to 1.38)
Men	349	175	1.12 (0.92 to 1.37)	0.83 (0.59 to 1.17)	1.28 (1.00 to 1.64)	349	175	1.29 (1.05 to 1.57)	1.61 (1.04 to 2.50)	1.19 (0.94 to 1.49)
Women	322	161	1.14 (0.94 to 1.38)	1.08 (0.82 to 1.43)	1.19 (0.91 to 1.55)	322	161	1.19 (0.97 to 1.45)	1.27 (0.91 to 1.78)	1.14 (0.88 to 1.47)
Surgery <5 years	148	74	0.96 (0.69 to 1.34)	0.81 (0.50 to 1.33)	1.03 (0.64 to 1.65)	148	74	1.57 (1.21 to 2.05)	1.97 (1.24 to 3.15)	1.34 (0.99 to 1.81)
Surgery ≥5 years	523	262	1.17 (1.00 to 1.37)	1.03 (0.80 to 1.31)	1.27 (1.05 to 1.55)	523	262	1.06 (0.88 to 1.28)	1.01 (0.68 to 1.50)	1.08 (0.86 to 1.34)

Values are the ORs with (95% CIs) for 1 (ln) SD increase (z-score) in the hs-CRP level or the hs-TnT level, as indicated. Z-scores with missing values replaced were used for all univariable calculations except when indicated.

Stratification for presence of CAD (yes/no), sex (men and women) and time between survey and surgery (less or more than 5 years).

Cut-offs for the quartiles (Q1–Q4) were: hs-CRP (mg/L; men and women): 0.5, 1.0, 2.5 and 0.6, 1.2, 2.3, respectively.

hs-TnT (ng/L; men and women combined) were 3.0, 3.7, 5.4. P values for indicating a trend: 0.026 for hs-CRP and 0.006 for hs-TnT.

CAD, coronary artery disease; hs-CRP, high-sensitivity C reactive protein; hs-TnT, high-sensitivity troponin T.

In multivariable analyses, elevated hs-TnT remained associated with surgery for AS after adjustment for hs-CRP, traditional risk factors and BMI in separate models (table 3). After stratification for sex and time between survey and surgery, the associations remained in men and in those with surgery within 5 years after survey. After stratification for CAD, elevated hs-TnT remained associated with AVR irrespective of CAD. Elevated hs-CRP levels did not associate with AVR after adjustments for cardiovascular risk factors. Exclusion of cases from the MSP cohort and their matched referents did not alter the results (data not shown).

**Table 3:** Multivariable analyses

		hs-CRP			hs-TnT		
		All, OR (95% CI)	No CAD, OR (95% CI)	CAD, OR (95% CI)	All, OR (95% CI)	No CAD, OR (95% CI)	CAD, OR (95% CI)
All	M1	1.11 (0.96 to 1.27)	0.98 (0.78 to 1.21)	1.21 (1.01 to 1.45)	1.22 (1.06 to 1.41)	1.39 (1.06 to 1.82)	1.14 (0.96 to 1.35)
	M2	1.06 (0.92 to 1.23)	0.91 (0.72 to 1.14)	1.13 (0.94 to 1.37)	1.26 (1.09 to 1.46)	1.39 (1.05 to 1.84)	1.22 (1.02 to 1.46)
	M3	1.05 (0.90 to 1.22)	0.88 (0.68 to 1.12)	1.13 (0.93 to 1.37)	1.24 (1.06 to 1.44)	1.35 (1.01 to 1.80)	1.21 (1.01 to 1.46)
Men	M1	1.09 (0.89 to 1.33)	0.83 (0.58 to 1.18)	1.25 (0.97 to 1.60)	1.27 (1.04 to 1.56)	1.62 (1.04 to 2.53)	1.15 (0.91 to 1.44)
	M2	1.06 (0.86 to 1.31)	0.70 (0.48 to 1.02)	1.21 (0.93 to 1.59)	1.36 (1.10 to 1.68)	1.56 (1.00 to 2.43)	1.31 (1.02 to 1.68)
	M3	1.06 (0.85 to 1.30)	0.67 (0.45 to 1.00)	1.21 (0.92 to 1.59)	1.37 (1.10 to 1.70)	1.51 (0.93 to 2.45)	1.32 (1.03 to 1.70)
Women	M1	1.12 (0.93 to 1.36)	1.08 (0.82 to 1.43)	1.17 (0.89 to 1.53)	1.18 (0.96 to 1.44)	1.27 (0.90 to 1.78)	1.12 (0.87 to 1.45)
	M2	1.08 (0.88 to 1.32)	1.04 (0.76 to 1.41)	1.11 (0.83 to 1.47)	1.20 (0.97 to 1.48)	1.29 (0.89 to 1.86)	1.18 (0.90 to 1.56)
	M3	1.03 (0.83 to 1.29)	1.04 (0.73 to 1.47)	1.05 (0.77 to 1.44)	1.15 (0.92 to 1.43)	1.24 (0.85 to 1.80)	1.14 (0.85 to 1.52)
Surgery <5 years	M1	0.91 (0.64 to 1.28)	0.80 (0.46 to 1.39)	0.96 (0.59 to 1.55)	1.58 (1.21 to 2.05)	1.95 (1.24 to 3.08)	1.34 (0.99 to 1.82)
	M2	0.97 (0.69 to 1.36)	0.86 (0.51 to 1.44)	1.07 (0.63 to 1.84)	1.65 (1.25 to 2.18)	2.05 (1.24 to 3.41)	1.53 (1.03 to 2.28)
	M3	0.96 (0.67 to 1.36)	0.74 (0.39 to 1.43)	1.06 (0.60 to 1.88)	1.66 (1.24 to 2.21)	2.01 (1.13 to 3.56)	1.58 (1.06 to 2.35)
Surgery ≥5 years	M1	1.17 (1.00 to 1.36)	1.02 (0.80 to 1.31)	1.27 (1.04 to 1.55)	1.04 (0.86 to 1.26)	1.01 (0.68 to 1.50)	1.03 (0.82 to 1.29)
	M2	1.09 (0.93 to 1.28)	0.93 (0.71 to 1.21)	1.16 (0.94 to 1.43)	1.04 (0.85 to 1.27)	0.89 (0.58 to 1.37)	1.09 (0.86 to 1.37)
	M3	1.08 (0.91 to 1.27)	0.94 (0.70 to 1.26)	1.14 (0.92 to 1.42)	1.01 (0.82 to 1.24)	0.85 (0.54 to 1.35)	1.06 (0.84 to 1.35)

Values are the ORs (95% CIs) for 1 (ln) SD increase (z-score) in the hs-CRP and hs-TnT levels, as indicated. Model 1 (M1) includes hs-CRP and hs-TnT; model 2 (M2) includes hs-CRP or hs-TnT plus glucose intolerance (yes/no), hypertension (yes/no), smoking (present or past/never), and Apo B/A1 ratio; model 3 (M3) includes model 2 plus BMI. Stratification for presence of CAD (yes/no), sex (men and women) and time between survey and surgery (less or more than 5 years).

Apo A1, Apolipoprotein A1; Apo B, Apolipoprotein B; BMI, body mass index; CAD, coronary artery disease; hs-CRP, high-sensitivity C reactive protein; hs-TnT, high-sensitivity troponin T.

**Figure 2:**
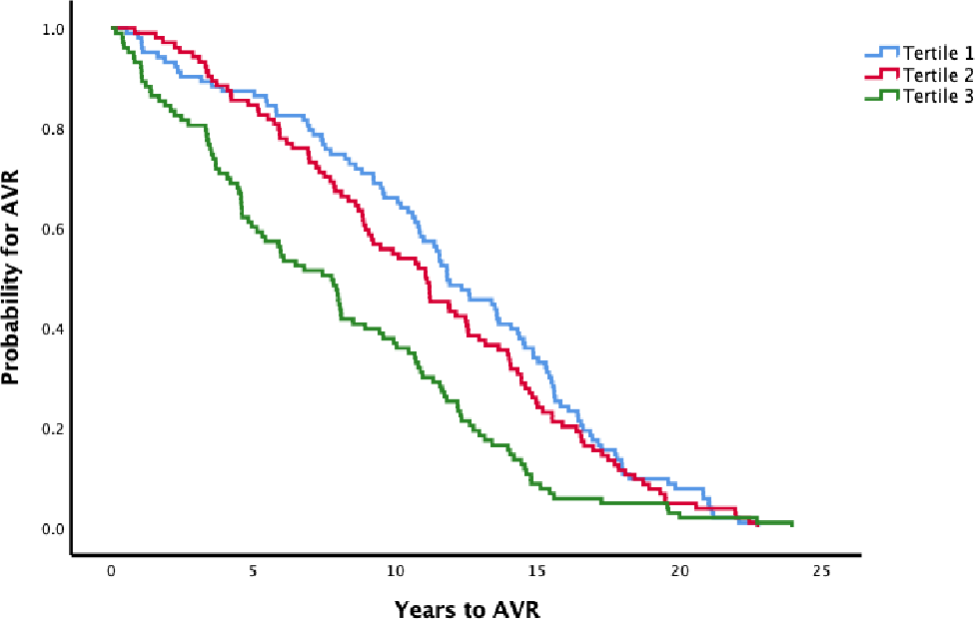
Time to surgery (AVR) stratified for tertiles of hs-TnT at the baseline survey. log-rank test; p<0.001. Cut-offs for tertiles; 3.2 and 5.1 ng/L. AVR, aortic valve replacement; hs-TnT, high-sensitivity troponin T.

**Figure 3:**
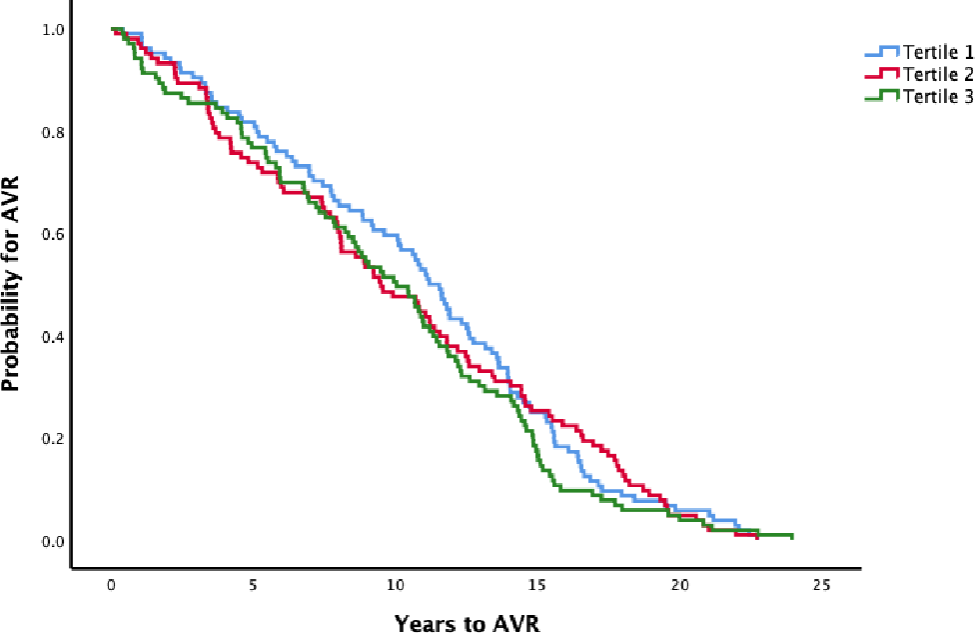
Time to surgery (AVR) stratified for tertiles of hs-CRP at the baseline survey. log-rank test; p=0.6. Cut-offs for tertiles; 0.8 and 2.1 mg/L. AVR, aortic valve replacement; hs-CRP, high-sensitivity C reactive protein.

Patients with elevated hs-TnT levels (tertile 3, see figure 2) had shorter time (median (95% CI)) to surgery compared with those with low levels (7.8 (5.9 to 9.6) vs 11.8 (10.1 to 13.6)) years, p<0.001), independently of sex and age at survey (HR 1.59 (1.15 to 2.21). In contrast, elevated hs-CRP levels (tertile 3, see figure 3) did not associate with time to surgery (10.0 (8.2 to 11.8) vs 11.2 (10.1 to 12.3)) years, p=0.6 and HR 1.11 (0.85 to 1.47)).

Hs-TnT levels at survey associated independently with myocardial mass at surgery (standardised beta=0.46, p=0.002) but not with gradient over the aortic valve (standardised beta=1.32, p=0.1), adjusted for sex (standardised beta=−024, p<0.001), age at survey (standardised beta=0.33, p<0.001) and time between survey and AVR sex (standardised beta=−029, p<0.001). The interaction term between left ventricular mass and gradient over the aortic valve did not reach significance (p=0.07). After stratification for sex, the association between hs-TnT levels at survey and left ventricular mass remained in both men and women (data not shown).

Hs-CRP levels at survey did not independently associate with myocardial mass at surgery (standardised beta=0.32, p=0.06), or with gradient over the aortic valve (standardised beta=1.73, p=0.07) in the fully adjusted model, adjusted for sex (standardised beta=0.07, p=0.3), age at survey (standardised beta=0.23, p=0.001) and time between survey and AVR sex (standardised beta=0.04, p=0.5). The interaction term between left ventricular mass and gradient over the aortic valve did not reach significance (p=0.08). Stratification for sex did not add more information.

## Discussion

In this nested, case-referent study, we showed that elevated baseline levels of hs-TnT independently associated with future AVR irrespective of CAD. In contrast, elevated hs-CRP levels did not associate with future AVR. Furthermore, hs-TnT associated with left ventricular mass at surgery, whereas hs-CRP did not.

### Biomarkers and AS

A number of serum biomarkers in valvular heart disease have been suggested.

The only biomarker that have been widely used in patients with AS is natriuretic peptides, reflecting myocardial strain, although their role in clinical management decisions is not yet clearly defined.^22–24^ Since the natural course of AS is progressive development of clinical symptoms that finally leads to heart failure and death, it is important to better understand the factors that determine the rate of progression which will help us to define optimal timing for interventions. Further, since many patients with AS remain asymptomatic for long periods of time, it is important to identify those patients that will develop complications. An ideal biochemical marker should help to identify baseline disease activity, predict progression of the disease and aid in the identification of high-risk patients in need of AVR.

As hs-TnT and hs-CRP reflect different pathophysiological mechanisms, and by contrasting them we hypothesised that the associations eventually found should reflect these different mechanisms.

### Hs-TnT and AS

With modern hs-TnT assays, it is possible to detect circulating TnT levels that are more than 10-fold lower than the lowest detectable level with previous methods. Accordingly, the majority of patients with stable CVD,^25^ as well as those with subclinical CVD,^26^ have detectable TnT levels, and elevated levels are associated with increased risk for all-cause and cardiovascular death, even in the absence of MI.^9^

To our knowledge, the impact of elevated hs-TnT years before surgery for AS has not been studied. Notably, the association between elevated hs-TnT and AVR was independent of CAD, and it was seen in those with less than 5 years between the baseline survey and AVR. This indicates that the progressing stenotic process induces pressure overload. This finding was further supported by the linear regression analysis done in cases only showing an association between elevated hs-TnT levels at survey and preoperative myocardial mass independently of the preoperative gradient over the aortic valve. This analysis also showed that patients with higher age and shorter time to AVR had higher hs-TnT levels at survey, and that female patients had lower hs-TnT probably reflecting generally lower myocardial mass in women.^27^ Also, the survival analysis showed that elevated hs-TnT levels were associated with shorter time to AVR.

More studies are warranted exploring if elevated hs-TnT could determine the appropriate timing for AVR.

We acknowledge that the hs-TnT levels indicating increased risk for AVR are very low (see table 2) and the clinical usefulness at this stage as a management tool is uncertain. However, troponins are attractive as biomarkers as the intraindividual variation is low over time,^28^ and appropriate levels for action should be determined in future studies.

### Hs-CRP and AS

Elevated levels of CRP reflect unspecific inflammation. The exact function of CRP is not yet fully understood but it is believed to be a part of the innate immune system.^29^ Galante *et al* demonstrated in 2001 that patients with severe degenerative AS (absence of CAD) had higher CRP level than controls.^30^ Further studies have shown divergent results.^11–13 31^ In this study, we did not find that elevated hs-CRP levels independently associated with neither increased risk for or time to AVR, nor with preoperative estimated myocardial mass.

Hs-CRP is, thus, probably a marker for cardiovascular risk factors but appears to be a poor predictor of subclinical AS.

### Limitations and strengths

This study has some limitations. CAD was treated as a categorical value (yes/no) that is, the burden of atherosclerosis was not considered. The matched design precludes us from studying the impact of matched factors on the risk for future surgery, and it is not possible to formally test if the risk related to (hs-CRP and) hs-TnT differs between men and women, except for showing the point estimates after stratification for sex. The baseline examination in the surveys (VIP, MONICA and MSP) did not include any imaging of the heart, and subclinical AS at survey in cases and in referents can thus not be excluded. Even if this study includes a comparably large numbers of patients, the power to detect associations of smaller magnitudes was limited, that is, the associations between hs-CRP and myocardial mass (see above).

The inclusion criteria for the VIP and MSP surveys determine the age distribution of the patient population and might have caused an under-representation of younger patients. Our cases are only patients accepted for AVR; thus, patients with contraindications for surgery, as well as asymptomatic patients, are not included (unless the primary indication for surgery was coronary by-pass surgery). Most patients had only one health survey with blood sampling before surgery and serial measurements of hs-TnT was thus not possible. Several other biomarkers including natriuretic peptides are of potential value but are lacking in this dataset. A more precise measurement of myocardial hypertrophy would have been preferable, but MRI of the heart was introduced lately. However, considering the large number of cases, the echocardiographic measurements used for the calculation of myocardial hypertrophy should be usable on the group level.

However, this study has some unique strengths that should be emphasised. First, the nested case-referent design which is truly prospective although cases are identified retrospectively. Second, the relatively large size of the cohort and the careful validation of each case which allowed us to stratify patients into clinically meaningful phenotypes. Finally, the standardised procedure of blood sample collection and that all blood samples from both cases and their matched referents were analysed at the same laboratory at a defined period of time. These procedures reduce both preanalytical and analytical biases that are often encountered in large prospective studies.

### Conclusion

Elevated plasma levels of hs-TnT were independently associated with increased risk and time to future surgery for AS. The association was seen in patients with shorter time to surgery. This finding has not been demonstrated previously and indicates that the myocardium is subject to mechanical stress already when the stenotic process is asymptomatic This may be used as a clinical tool and allow for earlier identification of patients with subclinical AS who potentially could benefit from earlier intervention. In contrast, elevated hs-CRP levels did not associate with risk for or time to future AVR. We conclude that hs-TnT and hs-CRP reflects different pathophysiological mechanisms related to AS and that hs-TnT could be a potential biomarker for determining intervention.
