# Supplementary material for: Troponin T but not C reactive protein is associated with future surgery for aortic stenosis: a population-based nested case-referent study
Source: Open Heart. 2020 Oct 13;7(2):e001325. doi: 10.1136/openhrt-2020-001325 (PMC7555075; doi:10.1136/openhrt-2020-001325)
Supplement: Supplementary data [file openhrt-2020-001325supp001.pdf]

Troponin T but not C–reactive protein is associated with future surgery for aortic stenosis - a population based nested case-referent study

Supplementary Table 1

Anders Holmgren[1], Johan Ljungberg[2], Johan Hultdin[3], Bengt Johansson[2], Ingvar A. Bergdahl[4], Ulf Näslund[2], and Stefan Söderberg[2].

Academic affiliation

[1]Department of Public Health and Clinical Medicine, Cardiac Surgery, Umeå University, Sweden

[2]Department of Public Health and Clinical Medicine, Medicine, Umeå University, Sweden

[3]Department of Medical Biosciences, Clinical Chemistry, Umeå University, Sweden

[4]Department of Biobank Research, Umeå University, Sweden

Corresponding author: Stefan Söderberg, Department of Public Health and Clinical Medicine, Umeå University, SE-901 87 Umeå, Sweden. Phone +46 90 785 18 46. E-mail stefan.soderberg@umu.se

Supplementary table 1. Preoperative characteristics

	Available no	No CAD	CAD	*P*
N (%)		132 (39.4)	203 (60.6)	
History				
Female sex (%)	132/203	59.1 (50.6–67.6)	40.9 (34.1–47.7)	0.001
Age at survey (years)	132/203	54.2 (52.5–55.8)	58.4 (57.3–59.4)	<0.001
Age at surgery (years)	132/203	63.8 (62.0–65.5)	69.6 (68.6–70.6)	<0.001
Known heart failure (%)	116/185	6.0 (1.6–10.4)	6.5 (2.9–10.1)	0.88
Known renal failure (%)	131/202	4.6 (1.0–8.2)	7.4 (3.8–11.1)	0.28
Previous MI (%)	126/198	1.6 (0–3.8)	13.1 (8.4–17.9)	<0.001
Previous CABG (%)	131/202	0.0	6.4 (3.0–9.8)	<0.001
Clinical parameters				
Systolic BP (mmHg)	128/195	139 (136–143)	144 (141–147)	0.05
Diastolic BP (mmHg)	127/195	81 (79–83)	82 (80–83)	0.61
Heart rate (bpm)	126/200	71 (69–74)	72 (70–74)	0.83
Preoperative echocardiography				
Root of aorta (mm)	128/191	34.1 (33.2–35.0)	34.0 (33.3–34.7)	0.83

Holmgren A, *et al. Open Heart* 2020; 7:e001325. doi: 10.1136/openhrt-2020-001325

Left atrium (mm)	126/189	40.7 (39.4–42.0)	42.1 (41.1–43.2)	0.10
LVEDD (mm)	130/195	50.2 (48.9–51.5)	49.5 (48.7–50.4)	0.38
LVESD (mm)	102/147	31.7 (30.2–33.2)	31.5 (30.2–32.8)	0.88
Intraventricular septum (mm)	117/170	13.0 (12.6–13.5)	13.2 (12.7–13.8)	0.59
Posterior wall (mm)	107/153	11.4 (11.0–11.8)	11.0 (10.7–11.3)	0.12
LV mass (g)	105/153	247 (230–264)	232 (221–243)	0.13
EF according to Teichholz (%)	103/147	66 (64–69)	65 (63–68)	0.62
LV function (reduced vs normal)	129/193	11.6 (6.0–17.2)	19.7 (14.0–25.3)	0.05
Aorta gradient max (mmHg)	132/196	78.9 (74.5–83.4)	68.0 (64.3–71.6)	<0.001
Aorta gradient mean (mmHg)	128/194	49.1 (46.0–52.1)	41.8 (39.3–44.2)	<0.001
VTI LVOT (cm)	110/162	22.2 (21.3–23.2)	21.7 (20.8–22.7)	0.50
VTI Aorta (cm)	107/159	105.4 (101.1–109.7)	94.0 (90.4–97.6)	<0.001
Aorta valve area (cm_2)	127/200	0.78 (0.74–0.81)	0.85 (0.81–0.89)	0.01

Data shown are findings from the preoperative assessment, stratified the presence of CAD according to the preoperative coronary angiogram. Available numbers and means with 95% confidence intervals are presented. All cases except one had a preoperative angiogram. CAD indicates coronary artery disease, MI myocardial infarction, CABG coronary by–pass grafting, BP blood pressure, LVEDD left ventricular end–diastolic diameter, LVESD left ventricular end–systolic diameter, LV left ventricle, VTI velocity time integral, LVOT left ventricle outflow tractus, and EF ejection fraction.

Holmgren A, *et al. Open Heart* 2020; 7:e001325. doi: 10.1136/openhrt-2020-001325
